# How Does the Delta-Radiomics Better Differentiate Pre-Invasive GGNs From Invasive GGNs?

**DOI:** 10.3389/fonc.2020.01017

**Published:** 2020-07-16

**Authors:** Yanqing Ma, Weijun Ma, Xiren Xu, Fang Cao

**Affiliations:** ^1^Zhejiang Provincial People's Hospital, Hangzhou, China; ^2^Shaoxing City Keqiao District Hospital of Traditional Chinese Medicine, Shaoxing, China

**Keywords:** ground-glass nodule, adenocarcinoma, invasive, radiomics, delta-radiomics, computed tomography

## Abstract

**Purpose:** This study aimed to explore the role of delta-radiomics in differentiating pre-invasive ground-glass nodules (GGNs) from invasive GGNs, compared with radiomics signature.

**Materials and Methods:** A total of 464 patients including 107 pre-invasive GGNs and 357 invasive GGNs were embraced in radiomics signature analysis. 3D regions of interest (ROIs) were contoured with ITK software. By means of ANOVA/MW, correlation analysis, and LASSO, the optimal radiomic features were selected. The logistic classifier of radiomics signature was constructed and radiomic scores (rad-scores) were calculated. A total of 379 patients including 48 pre-invasive GGNs and 331 invasive GGNs with baseline and follow-up CT examinations before surgeries were enrolled in delta-radiomics analysis. Finally, the logistic classifier of delta-radiomics was constructed. The receiver operating characteristic curves (ROCs) were built to evaluate the validity of classifiers.

**Results:** For radiomics signature analysis, six features were selected from 396 radiomic features. The areas under curve (AUCs) of logistic classifiers were 0.865 (95% CI, 0.823–0.900) in the training set and 0.800 (95% CI, 0.724–0.863) in the testing set. The rad-scores of invasive GGNs were larger than those of pre-invasive GGNs. As the follow-up interval went on, more and more delta-radiomic features became statistically different. The AUC of the delta-radiomics logistic classifier was 0.901 (95% CI, 0.867–0.928), which was higher than that of the radiomics signature.

**Conclusion:** The radiomics signature contributes to distinguish pre-invasive and invasive GGNs. The rad-scores of invasive GGNs were larger than those of pre-invasive GGNs. More and more delta-radiomic features appeared to be statistically different as follow-up interval prolonged. Delta-radiomics is superior to radiomics signature in differentiating pre-invasive and invasive GGNs.

## Introduction

Pulmonary nodules are one of the most common incidental findings (1). Ground-glass nodule (GGN) is a distinct subgroup of pulmonary nodules, which is a complex diagnostic challenge, including a broad array of benign and malignant lesions (2). GGN is defined as a hazy shadow presenting intact bronchial structures and pulmonary vessels (3), which is generally associated with early-stage lung adenocarcinoma. The lung adenocarcinomas are classified into three histological subtypes, namely, adenocarcinoma *in situ* (AIS), minimal invasive adenocarcinoma (MIA), and invasive adenocarcinoma (IAC), according to the International Association for the Study of Lung Cancer/American Thoracic Society/European Society of Thoracic Surgeons classification (4). Some benign GGNs can also be observed, such as interstitial fibrosis, inflammation, hemorrhage, and atypical adenomatous hyperplasia (AAH) (5).

It is the malignant potential and aggressive characteristics that make the diagnosis of GGN challenging for radiologists. Generally, pre-invasive GGNs include AAH and AIS, while MIA and IAC are categorized into invasive GGNs (6). Different histopathological types of GGNs have different growth and invasive speeds. The continuous process from AAH to IAC has been proposed (7), showing the increase of diameter and density in GGNs (8). The 5-years survival rate has been reported to be almost 100% for early-stage lung cancer patients, who were diagnosed as AAH and AIS. However, the 5-years survival rate of patients with IAC is only 60–70% (9). Therefore, early differentiation between pre-invasive and invasive GGNs is important for clinical management.

The natural chronologic evolution of GGNs on CT scans remains to be elucidated. Though the Fleischner Society published the recommendations for management of subsolid nodules and updated guidelines based on the latest data and accumulated opinions from a multidisciplinary international group (10). Both radiologists and pulmonologists are confronted with the dilemma of choosing the most adequate diagnostic scheme and optimal management strategies for GGNs. Therefore, it is difficult to determine proper follow-up examinations, due to different growth patterns of GGNs.

Radiomic analysis is a newly emerging computer-assisted approach, converting conventional visual images into numerous quantitative features (11). The features covered voxel intensity, three-dimensional shape, size, appearance of surface, and the gray level co-occurrence. It has been widely employed in the differentiation and diagnosis of breast lesions (12), renal neoplasms (13), liver disease (14), and brain tumors (15) on CT examination or magnetic resonance imaging (16). Several studies have also attempted to elaborate on the radiomic characteristics of pulmonary GGNs (17). Jing et al. developed computer-aided radiomic analysis to improve the performance in discriminating different subtypes of GGO nodules (18). The current study found that radiomics signature showed good predictive performance in differentiating IACs and non-invasive lesions (19). Moreover, delta-radiomics analysis shows the changes in radiomics features between baseline and follow-up examinations, during treatment, and so on. It has been demonstrated that delta-radiomic features combined with conventional radiomic features improved performance of models in lung cancer screening (20).

To the best of our knowledge, there are no published studies focused on delta-radiomics in differentiating pre-invasive and invasive GGNs using 3D CT images. The purpose of our study is to evaluate the progressive changes of delta-radiomics CT analysis to differentiate pre-invasive and invasive GGNs, compared with radiomics signature.

## Materials and Methods

### Patient Selection

This retrospective study was approved by the institutional review board of our hospital, which waived the written informed consent.

Between January 2015 and August 2019, there were 391,985 chest CT scan examinations carried out in our institution and 195,238 cases diagnosed referring to pulmonary lesions. A total of 2,064 patients were histopathologically confirmed after surgical resections or CT-guided percutaneous biopsies. After reviewing all the images of 2,064 cases, 464 patients were eventually enrolled in our study. The inclusion criteria for the selected GGNs were as follows: (a) CT examinations were performed with the same acquisition protocol; (b) histopathological diagnosis was made after surgical resection; (c) the diameter of all GGN was smaller than 3 cm in CT images; (d) there was a single solitary lesion in the lung; (e) patients received the same thin-section CT scans, with a slice thickness of 2.0 mm. The exclusion criteria were as follows: (a) patients had malignant tumor history; (b) patients had multiple pulmonary lesions, such as interstitial pneumonia, pulmonary infection, chronic obstructive pulmonary disease, and so on; (c) the histopathological diagnosis was not lung adenocarcinoma; (d) patients underwent neoadjuvant chemotherapy or radiotherapy; (e) patients were diagnosed by biopsy (Table 1).

**Table 1:**
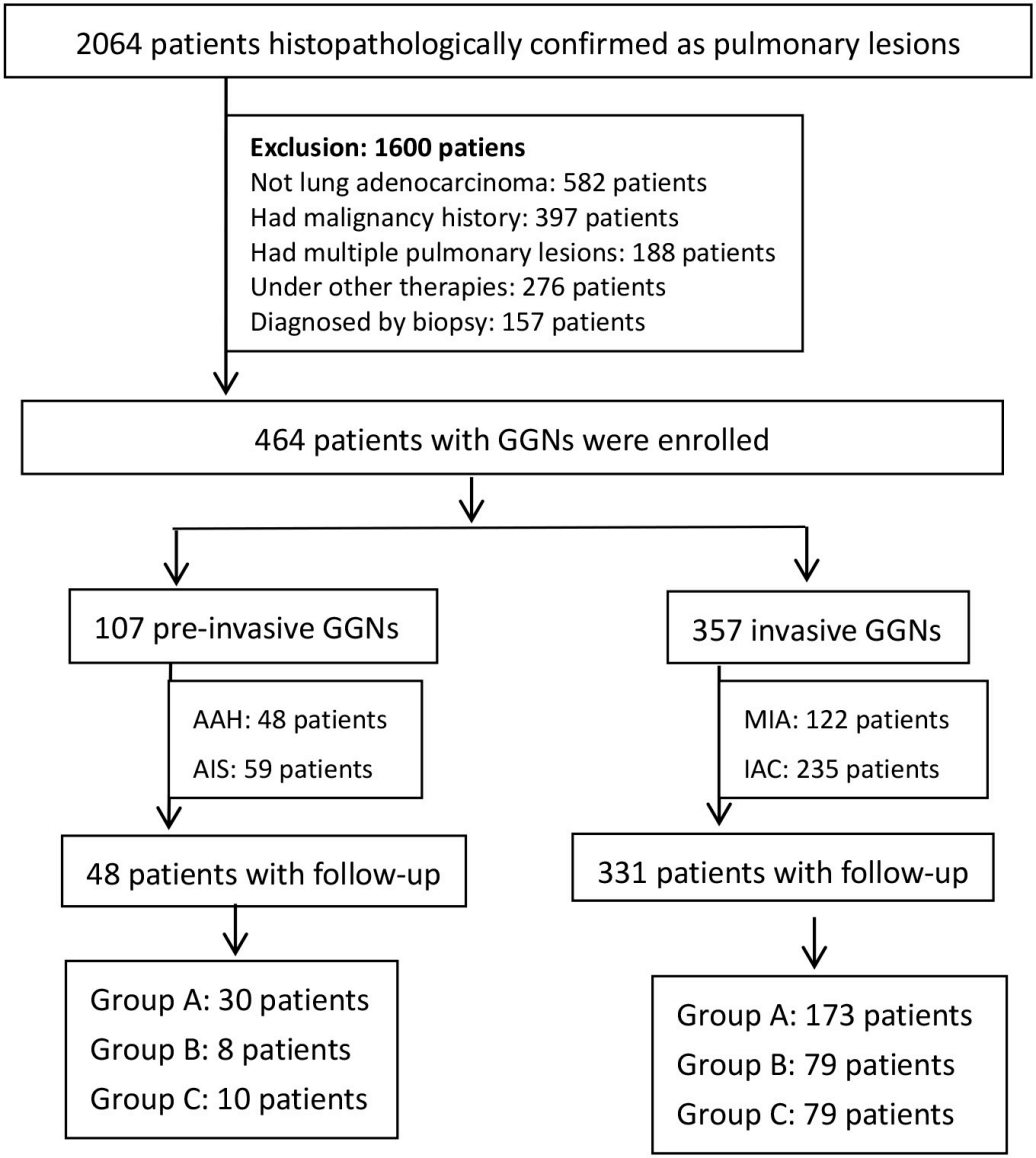
The flowchart of patient selection.

According to histopathological diagnosis, the enrolled 464 patients were divided into 107 pre-invasive GGNs (48 patients with AAH, 59 patients with AIS) and 357 invasive GGNs (122 patients with MIA, 235 patients with IAC).

### CT Image Acquisition

All patients were examined by Somaton Definition AS 64/128 (Siemens Medical Solutions, Germany). Patients performed the CT scan in the supine position from the apex to the lever of adrenal glands during inspiration. The scan parameters were as follows: slice thickness and reconstruction interval, 2.0 mm; tube voltage of 120 kVp and tube current of 200 mA; detector collimation, 64^*^0.625 mm; rotation speed, 0.75 s; beam pitch, 1.375; pixel matrix, 512^*^512. The CT images were reconstructed with a bone algorithm for the lung window and a soft tissue algorithm for the mediastinal window. The same lung window (width, 1,500 HU; level, −600 HU) and mediastinal window (width, 350 HU; level, 50 HU) were adopted to assess the images.

GGNs delta-radiomic features were calculated as the change of radiomic features from baseline CT scans to the final follow-up CT scans before surgeries and then divided by the time interval ([follow-up time – baseline time]/30) in both pre-invasive and invasive GGNs (delta-radiomics = [follow-up radiomics – baseline radiomics]/time interval).

### Region of Interest (ROI) Segmentation and Radiomics Signature Analysis

**Figure 1:**
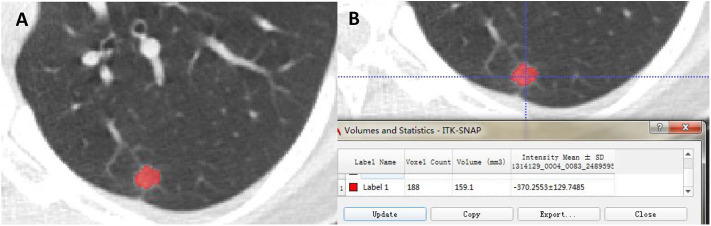
The ROI was semi-automatically delineated using the software “ITK-SNAP” **(A)**. The volume and intensity of GGN were calculated subsequently **(B)**.

The radiologists with 10 and 15 years of CT diagnosis experience manually delineated ROIs of all the images independently and the intra-class correlation coefficient (ICC) was calculated. The data from two radiologists after discussing by consensus or adjudication was adopted, ultimately. ROIs were manually depicted in 3D CT images using the software “ITK-SNAP” (Version 3.4.0, www.itksnap.org), keeping an ~2–3 mm distance away from the lesion margin to minimize the partial volume effect (Figure 1A). The volume and mean intensity of 3D GGN were calculated automatically (Figure 1B).

The radiomic features were analyzed by AK software (Artificial Intelligence kit V3.0.0.R, GE Healthcare), including histogram, texture, form factor, gray level co-occurrence matrix (GLCM), and gray level run-length matrix (RLM). Prior to analysis, three preprocessing steps were taken to normalize images, including resampling with 1.0 mm at *X*/*Y*/*Z*-spacing, denoising by Gaussian, and discretizing the gray level from 0.0 to 255.0. Then, we calculated radiomic features by AK software, automatically.

Four steps were needed to reduce radiomic dimensions: First, replacing the abnormal values by mean and standardization. Second, partitioning the training and testing data with a proportion of 7:3, randomly. Third, after the normality test, analysis of variance (ANOVA) or Mann–Whitney *U* test (MW) was used to select the radiomic features. Fourth, set the filter threshold of 0.9 for the Spearman rank correlation coefficient analysis to reduce the dimensions. Ultimately, use the Least Absolute Shrinkage and Selection Operator (LASSO) Cox regression model to identify the optimal features. The logistic classifier of radiomics signature was constructed and the rad-scores of pre-invasive and invasive GGNs were calculated. The predictive accuracy of radiomics signature was quantified by ROCs in both training and testing sets. More information about radiomic dimensions and the LASSO algorithm can be found in the Supplementary Data.

### Delta-Radiomics Analysis

A total of 379 patients in the entire cohort of 464 patients were detected at baseline and follow-up CT examination before surgeries and were divided into three groups according to different time intervals: (a) group A: follow-up interval was <6 months; (b) group B: follow-up interval was between 7 and 12 months; (c) group C: follow-up interval was between 13 and 24 months. The significance of selected radiomic features in differentiating pre-invasive and invasive GGNs between three groups was evaluated. The changes of selected optimal radiomic features (delta-radiomic features) were calculated between baseline and follow-up. Multivariate logistic classifier of delta-radiomics was constructed to identify the predictive accuracy in distinguishing pre-invasive and invasive GGNs.

### Statistics

The methods of ANOVA/MW, Spearman rank correlation coefficient analysis and LASSO Cox regression were made by R software (Version 3.6.1) to select meaningful radiomic features. A paired Student's *t*-test was used if continuous variables were normally distributed; otherwise, Wilcoxon rank sum test was performed between different follow-up intervals in the delta-radiomics analysis by SPSS (IBM Statistics SPSS 22.0). The delta-radiomics classifier was modeled by means of multivariate logistic regression, and the ROC curve was depicted. The ROC curves of training/testing set in radiomics signature analysis and delta-radiomics analysis were made with MedCalc (Version 15.8). A *p*-value <0.05 was considered statistically significant.

## Results

### Patients' General Characteristics

The general characteristics of 464 patients are summarized in Table 2. Of the patients, 107 (23.1%) were categorized as pre-invasive GGNs (48 with AAH, 59 with AIS), and 357 (76.9%) as invasive GGNs (122 with MIA, 235 with IAC). Among the 107 pre-invasive GGNs patients, 74 (69.2%) patients were female (mean age, 51.3 ± 9.0 years) and 33 (30.8%) patients were male (mean age, 62.7 ± 10.4 years). Among the 357 invasive GGNs patients, 215 (60.2%) patients were female (mean age, 55.4 ± 13.0 years) and 142 (39.8%) patients were male (mean age, 62.3 ± 12.9 years).

**Table 2:** Patients' general characteristics.

**Patients' general characteristics**	**Pre-invasive GGNs**	**Invasive GGNs**	***p***
Gender (female/male)	74 (69.2%)/33 (30.8%)	215 (60.2%)/142 (39.8%)	0.058
Age	54.8 ± 10.8	58.1 ± 13.4	0.021
Lesion volume (mm^3^)	174.1 ± 253.9	841.2 ± 1380.8	<0.001
Intensity	−342.4 ± 135.6	−355.1 ± 127.0	0.372
Location (right/left)	69 (64.5%)/38 (35.5%)	209 (58.5%)/148 (41.5%)	0.162
Follow-up patients (group A/B/C)	48 (30/8/10)	331 (173/79/79)	/
Mean follow-up interval (months)	8.5	8.0	/

*The gender and location characteristics were compared by Chi-square test, while age, lesion volume, and intensity characteristics were compared by ANOVA. p < 0.05 has statistical significance*.

### Radiomic Feature Selection and Prediction of Radiomics Signature

**Figure 2:**
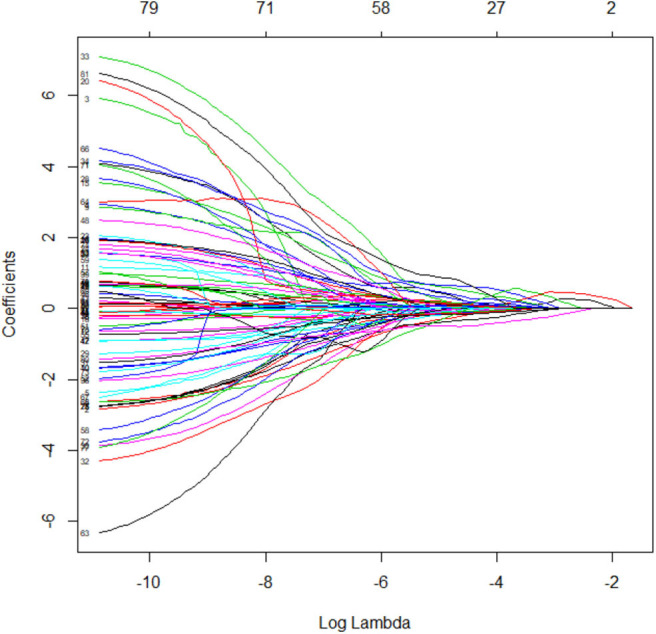
The LASSO coefficient profiles of radiomics signature.

**Figure 3:**
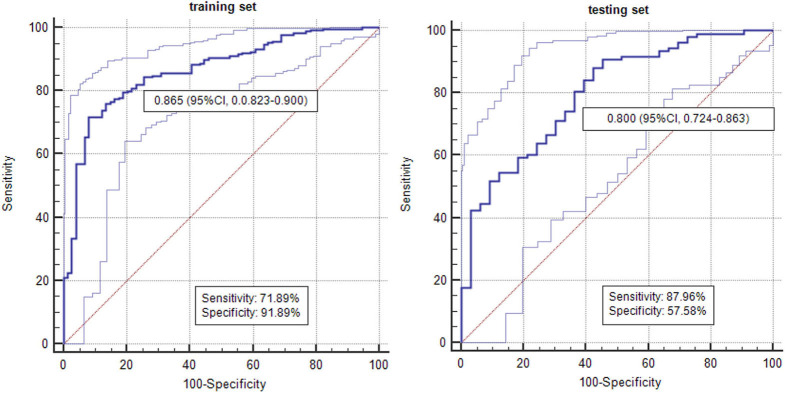
The AUCs of radiomics signature in differentiating pre-invasive and invasive GGNs in the training set and testing set.

**Figure 4:**
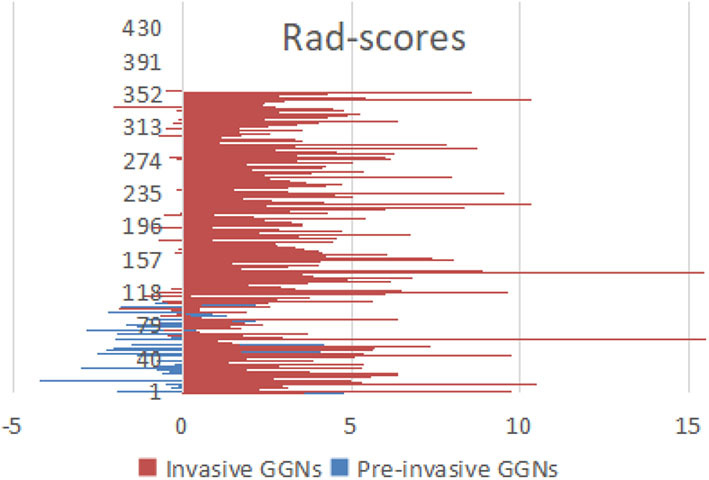
The rad-scores of invasive GGNs were larger than those of pre-invasive GGNs.

By means of ANOVA/MW, Spearman rank correlation coefficient, and LASSO Cox regression analysis, six features were selected from 396 radiomic features (Figure 2). The selected six features were standard deviation, inertia of GLCM, sum Entropy, high gray level run emphasis, size zone variability, and low-intensity small area emphasis. The AUCs of radiomics signature classifier were 0.865 (95% CI, 0.823–0.900) in the training set and were 0.800 (95% CI, 0.724–0.863) in the testing set (Figure 3). The rad-scores of invasive GGNs were larger than that of pre-invasive GGNs (Figure 4).

### Delta-Radiomics Analysis

Of the 107 pre-invasive GGNs patients, 48 patients were detected at baseline and follow-up CT examination before surgeries, including 30 patients in group A, 8 patients in group B, and 10 patients in group C. There was no statistical difference in the six selected radiomic features in group A, while sum entropy (*p* = 0.003) and size zone variability (*p* = 0.028) had a significant difference between baseline and follow-up examinations in group B. In group C, there was significant difference in standard deviation (*p* = 0.005), sum entropy (*p* = 0.005), high gray level run emphasis (*p* = 0.012), and size zone variability (*p* = 0.020) (Table 3).

**Table 3:** The delta-radiomic features in different follow-up intervals.

***p*****-value**		**Standard deviation**	**Inertia of GLCM**	**Sum entropy**	**High gray level**	**Size zone**	**Low-intensity**
					**run emphasis**	**variability**	**small-area emphasis**
Pre-invasive GGNs	Group A	0.843*	0.351	0.792*	0.627	0.402*	0.440
	Group B	0.103*	0.878*	0.003*	0.406*	0.028*	0.203*
	Group C	0.005*	0.235*	0.005*	**0.012**	0.020*	0.952*
Invasive GGNs	Group A	**0.007**	0.115	**0.009**	0.794	**0.013**	0.804
	Group B	**0.007**	**0.030**	**<0.001**	0.809	**<0.001**	0.867
	Group C	**<0.001**	0.007*	**<0.001**	0.931	**<0.001**	0.061

*Group A: follow-up interval <6 months; group B: follow-up interval of 7–12 months; group C: follow-up interval of 13–24 months*.

If continuous variables were normally distributed, Paired Student's t-test was used (^*^), while Wilcoxon rank sum test was performed. p < 0.05 had significant difference.

*Bold values signifies p < 0.05*.

There were 331 patients detected at baseline and follow-up CT examinations in the 357 invasive GGNs, including 173 patients in group A, 79 patients in group B, and 79 patients in group C. There were statistical differences among three radiomic features, namely, standard deviation (*p* = 0.007), sum entropy (*p* = 0.009), and size zone variability (*p* = 0.013) in group A, while there was statistical significance among four features, including standard deviation (*p* = 0.007, *p* = 0.000, respectively), inertia of GLCM (*p* = 0.030, *p* = 0.007, respectively), sum entropy (*p* = 0.000, *p* = 0.000, respectively), and size zone variability (*p* = 0.000, *p* = 0.000, respectively) in both group B and group C (Table 3).

**Figure 5:**
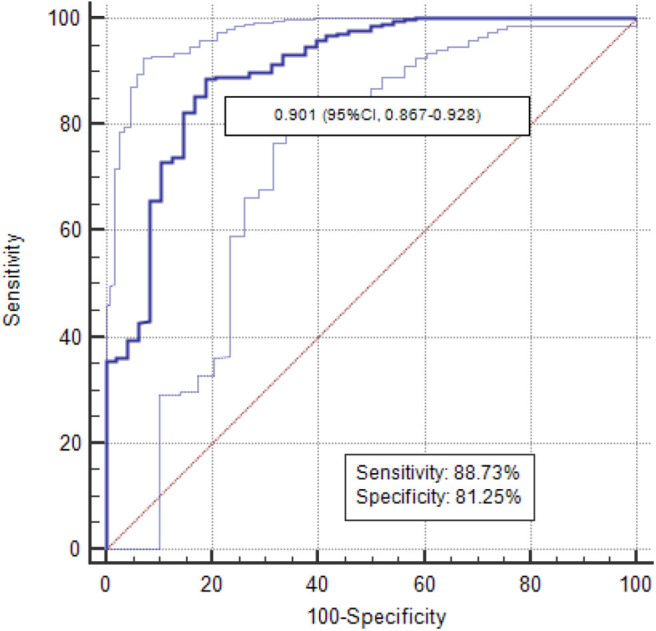
The AUCs of delta-radiomics logistic classifier was 0.901.

The delta-radiomic features between the baseline and final follow-up CT examinations were calculated. Multivariate logistic regression classifier of delta-radiomics in selected six features was built. The AUC of classifier was 0.901 (95% CI, 0.867–0.928) in differentiating pre-invasive from invasive GGNs (Figure 5).

### The Intra-Observer Agreement

The radiologists with 10 and 15 years of CT diagnosis experience delineated ROIs of all the images, respectively. The ICC was calculated to evaluate the intra-observer agreement of feature selection. The parameters of the selected six features from two radiologists were compared. The intra-observer ICC ranged from 0.782 to 0.913. ICC, which was >0.75, showed favorable reproducibility of feature selection between different observers.

## Discussion

With the development of CT examination, GGNs have been more frequently detected and became a major concern. Studies have proven that it may take several years in process from AAH to IAC by stepwise progression (21). Early diagnosis of GGNs has great therapeutic significance in patient management. How to identify pre-invasive GGNs and invasive GGNs remains a challenge for radiologists. Lee et al. concluded that the risk of GGNs' invasiveness gradually increased with the increase of maximal diameter (22). However, the optimal time of intervention based on the maximal diameter of GGN remains to be studied. In a previous study, a significant proportion of GGNs showed an indolent course for more than 2 years without size increase (23). Recent evidence suggests that GGNs have different natural histories, including growing up, shrinkage, or remaining stable for long periods (24). Therefore, visual evaluation of CT imaging characteristics is insufficient to differentiate pre-invasive GGNs from invasive GGNs. Radiomics signature as a new emerging quantitative method is necessary to reevaluate diagnostic performance in distinguishing pre-invasive and invasive GGNs.

In our study, we proposed a novel 3D radiomics signature analysis to classify GGNs, with an AUC of 0.865 in the training set and an AUC of 0.800 in the testing set. Most studies in either radiomic analysis or conventional CT characteristics analysis have only focused on 2D axial CT images previously. Meanwhile, our study evaluated the natural course of GGNs based on delta-radiomic features measured on 3D whole tumor. Due to the asymmetric growth pattern, 3D computer-aid analysis offers obvious advantages for accurate distinguishing. To avoid bias, we compared the six selected radiomic features from two radiologists. The intra-observer ICC, which ranged from 0.782 to 0.913, indicated favorable intra-observer agreement in feature extraction. Accordingly, questions have been raised about the low sensitivity, specificity, and AUC of conventional CT analysis in discriminating (6). It is becoming increasingly important to take radiomic analysis to monitor GGNs. We synchronized all the selected radiomic features into an indicator of rad-score. The rad-scores of invasive GGNs were higher than those of pre-invasive GGNs.

The Fleischner Society guidelines for the management of solid nodules were published in 2005, and separate guidelines for subsolid nodules were issued in 2013 (25). However, awareness and conformance to Fleischner guidelines vary considerably, and overmanagement or additional examinations are common (1). The management of pulmonary GGNs remains a challenge with some controversial issues. Tumor growth may be inconstant throughout the tumor's natural course as it reflects the expression of more aggressive elements (26). Nonetheless, single volume or intensity measurements at different follow-up time points are inadequate. Our study focuses on the delta-radiomics of GGNs from baseline to follow-up. For invasive GGNs, three radiomic features already have significant difference in group A with the follow-up interval of 0–6 months, while there is no significant difference in radiomic features for pre-invasive GGNs in group A. Two and four radiomic features have significant difference in group B for pre-invasive and invasive GGNs, respectively. Thus, as the follow-up interval goes on, more and more radiomic features become different. These results could assist in determining management and therapeutic strategies for both pre-invasive and invasive GGNs. Multivariate logistic regression analysis was used to evaluate the delta-radiomics in discriminating invasiveness of GGNs. The corresponding ROC curve was drawn to estimate the predictive accuracy of delta-radiomics logistic classifier. The delta-radiomics had higher AUC than radiomics signature in identifying invasive GGNs (0.901 vs. 0.865/0.800). It is important to note that follow-up examination is of great significance in distinguishing pre-invasive GGNs and invasive GGNs.

This study had several limitations. First, the follow-up intervals between two consecutive CT examinations were heterogeneous within 2 years. Obviously, the 2-years follow-up period is insufficient for GGNs. Second, we abandoned the GGNs that were followed-up without surgeries. This factor may give rise to selecting bias. Third, the small vessels located in the GGNs cannot be excluded during the segmentation process, though the vessels contiguous to lesion contours were removed manually. Fourth, multi-central prospective studies are necessary to confirm the conclusion in this study.

In conclusion, radiomics signature helps differentiate pre-invasive GGNs from invasive GGNs. The rad-scores of invasive GGNs are larger than those of pre-invasive GGNs. With the follow-up interval prolongs, the delta-radiomic features increase. The delta-radiomics analysis has a higher AUC than radiomics signature in identifying invasive GGNs.

## Data Availability Statement

The raw data supporting the conclusions of this article will be made available by the authors, without undue reservation, to any qualified researcher.

## Ethics Statement

The studies involving human participants were reviewed and approved by the ethics committee of Zhejiang Provincial People's hospital, and the written informed consent requirement was waived.

## Author Contributions

YM: conceptualization, methodology, writing-original draft, writing-review and editing, and supervision. WM: software, formal analysis, and data curation. XX: validation, resources, resources, and visualization. FC: validation and resources.

## Conflict of Interest

The authors declare that the research was conducted in the absence of any commercial or financial relationships that could be construed as a potential conflict of interest.
