# Supplementary material for: How Does the Delta-Radiomics Better Differentiate Pre-Invasive GGNs From Invasive GGNs?
Source: Front Oncol. 2020 Jul 16;10:1017. doi: 10.3389/fonc.2020.01017 (PMC7378390; doi:10.3389/fonc.2020.01017)
Supplement: Supplementary file 1 [file Data_Sheet_1.PDF]

Image preprocessing

we used z-score normalization to make the image intensities have the properties of a standard normal distribution with $\mu=1$ and $\sigma=0$, where μ was the mean value of the images, and σ was the standard deviation. The normalized values (also called z scores) of the image intensities (*x*) were calculated as follows:

$$z = \frac{x - \mu}{\sigma}$$
