# Supplementary material for: How Does the Delta-Radiomics Better Differentiate Pre-Invasive GGNs From Invasive GGNs?
Source: Front Oncol. 2020 Jul 16;10:1017. doi: 10.3389/fonc.2020.01017 (PMC7378390; doi:10.3389/fonc.2020.01017)
Supplement: Supplementary file 2 [file Data_Sheet_2.PDF]

Supplementary Material

1. The methodology of radiomic features reduction

Firstly, extracted radiomic features were standardized, which could remove the unit limits of the data of each feature, so that indexes of different units or orders could be compared and weighted.

Then the radiomic dimension reduction was performed as follows: (a) After normality test, analysis of variance (ANOVA) was used if continuous variables were normally distributed, otherwise Mann-whitney U-test was performed. (b) The correlation test was calculated to reduce data redundancy. The software calculated the paired correlation between each two radiomic features. Setting the filter threshold of 0.9. If the Spearman correlation coefficient was greater than 0.9, which showed that the two features were highly correlated, one of them was removed. All of the above steps were carried out by R statistical software.

2. The least absolute shrinkage and selection operator (LASSO) algorithm

LASSO is a powerful algorithm for regression analysis with high dimensional predictors. In our study, the LASSO algorithm was combined with the logistic regression model for model development. We used the LASSO logistic regression model to select the most important predictive features and construct a radiomics signature in the training set. This algorithm minimizes a log partial likelihood subject to the sum of the absolute values of the parameters bounded by a constant:

$$\hat{\beta} = arg\ min\ l(\beta) \text{, subject to } \sum|\beta_j| \leq t$$

where $\hat{\beta}$ is the obtained parameters, $l(\beta)$ is the log partial likelihood of the logistic regression model, and $t > 0$ is a constant.

The LASSO algorithm shrinks some coefficients and reduces others to exactly 0 via the absolute constraint. Thus, LASSO is an outstanding method for feature selection by retaining the good features of both subset selection and ridge regression. In this study, LASSO selected 2 nonzero coefficients $\hat{\beta}$, and a formula was generated using a linear combination of selected features that were weighted by their respective LASSO coefficients. The “glmnet” package in R

statistical software version 3.6.1 was used for LASSO logistic regression model analysis.
